# Barriers to Screening Pregnant Women for Domestic Violence: A Cross-Sectional Study

**DOI:** 10.4172/2471-9846.1000207

**Published:** 2018-01-17

**Authors:** Simon Nderitu Githui, Margaret Chege, Miriam CA Wagoro, James Mwaura

**Affiliations:** Department of Nursing, University of Eastern Africa Baraton, Eldoret, Kenya

**Keywords:** Barriers, Domestic violence, Screening, Pregnant women, Nurse

## Abstract

**Background information:**

Domestic Violence (DV) is associated with serious consequences to the survivor’s physical, emotional, sexual, social and mental well-being. DV screening ensures timely detection of violence and hence promotes timely intervention. This timely intervention has the potential of averting adverse outcomes of DV to the survivor. Globally, the prevalence of DV among women is 35% and in Kenya its 49% among women and 13.5% among pregnant women. Despite the adverse outcome of DV in pregnancy, screening during pregnancy lags behind in Kenya.

**Purpose:**

To assess the nursing barriers to screening pregnant women for DV.

**Methodology:**

A cross-sectional study of 125 nurses selected by random sampling method was conducted at a National Maternity Hospital in Kenya. Data was collected for 8 weeks using researchers developed structured questionnaire. Data was analyzed using Statistical Package for Social Sciences (SPSS) version 20.0. Chi-square test was used to determine significance of relationships between nominal variables. A P-value of ≤ 0.05 was considered significant.

**Results:**

Study results revealed that 16% (n=8) of nurses routinely screened pregnant women for DV. Non-screening behavior of nurses was associated with lack of DV screening training during their education program (P=0.002), fear of the partner’s reaction (P=0.004) and lack of mentors and role models in DV screening (P=0.005). Lack of cooperation from other health professionals was also associated with non-screening of DV (P=0.016).

**The significance of the study:**

The results of this study point to the need of developing hospital’s protocols on DV management and considering integrating DV screening in the routine medical screening of pregnant women during antenatal care.

**Conclusion:**

The study showed that the nurse’s prevalence of screening pregnant women for DV is low at 16% due to various barriers.

## Introduction

Domestic Violence (DV) is a serious, preventable public health problem that affects millions of women globally [1]. The prevalence of DV among pregnant women in Africa is 37% [2] compared to a worldwide prevalence of 35% [1] and 13.5% in Kenya [3,4]. The prevalence of DV in Kenya is higher than many conditions such as hypertension and anemia, which are routinely screened during pregnancy [5]. But there is a culture of silence surrounding gender-based violence, even women who want to speak about their experiences of domestic violence may find it difficult because of feelings of shame or fear [6].

Despite universal screening recommendations for DV [7], screening for DV in health care settings in general and during pregnancy in particular, is far from being implemented universally [1]. Research indicates that the prevalence of screening for DV is relatively low. Women are commonly not asked about DV when treated in most health facilities. This is despite the evidence that women experiencing violence often seek help in emergency departments [8]. On the other hand, midwives are concerned, interested and knowledgeable about DV screening. However, they screened less than half of the recommended time and did not use any standardized screening tool [9]. Also, as reported by Baig et al. [10], 95% of health care providers had adequate knowledge about DV screening, but only 15% of them routinely screened for DV.

DV, if not screened and managed, it is detrimental to the physical, emotional, sexual, social and mental well-being apart from being a violation of human rights [11,12]. It’s directly associated with negative health consequences to both the mother and the newborn to include maternal and newborn mortalities [13]. For instance, Ackerson and Subramanian [13] and Janssen [14] reported the link between DV and high risk of antepartum hemorrhage, intrauterine growth restriction and neonatal death. Without strategies to reduce prevalence of DV, achievement of Millennium Development Goals (MDGs) numbers 3, 4 and 5 that aim to promote gender equality and empowerment of women, reducing child mortality and improving of maternal health would be derailed.

Screening for DV has the potential to improve health outcomes for the women and their newborn. This is because it promotes early detection of violence and hence timely interventions, which are essential in averting adverse outcomes [15]. However, there is no consensus on the frequency of screening. Although some agencies recommend universal screening for DV [7,16], others recommend periodic and focused screening [17,18]. As a result of this, in most health care settings and during pregnancy in particular, screening for DV is far from being implemented [16,19,20]. In Kenya despite the recent evidence by Undie et al. [3,4] on high acceptability and feasibility of potential DV screening interventions, screening does not take place in most health care facilities. It is therefore important to document any barriers that may hinder the nurse’s role in screening pregnant women for DV if universal routine screening is to be achieved in Kenya.

## Materials and Methods

A cross-sectional descriptive study was conducted at the largest maternity hospital in Kenya. Stratified random sampling was utilized to select 125 nurses and midwives working in Antenatal Clinics (ANC), antenatal wards, labour wards and maternity theatre. Lottery method was used to sample participants from each stratum. The purpose of the study was explained to the nurses and midwives during weekly departmental meetings at the hospital. Memos inviting eligible nurses to participate in the study were posted on the hospital’s notice board 3 weeks prior to the commencement of the study. This was to create awareness and to give an opportunity to all those who were interested in the study to participate. During the 8 week period of data collection, the researchers were stationed at the nursing stations/desks to recruit eligible nurses as they completed their shifts. A semistructured questionnaire developed after a comprehensive review of literature was used to collect data. Screening was measured by a response indicating “I screen always” and “I screen most of the time” on the question: How often do you currently screen pregnant women for DV? A barrier was measured by a yes response on each specific item that was listed as an anticipated barrier. For categorical variables such as gender, level of education, years of practice, frequencies and percentages were computed. Data was further analysed using Statistical Package for Social Sciences (SPSS) version 20.0. Chi-square was used to determine significance of relationships between two nominal variables at 95% confidence interval and a P-value of ≤ 0.05 was considered significant. The study was approved by the University of Nairobi, Kenyatta National Hospital Ethics and Research Committee. Participants were required to give a signed, voluntary informed consent prior to participation in the study without coercion. The anonymity of participants was ensured by serializing the structured questionnaires.

## Results

A total of 125 nurses were recruited into the study, male respondents were 22.4% (n=28) and females were 77.6% (n=97). The majority of the respondents (38.4%, n=48) were between the age of 40 years and 49 years and only one nurse (0.8%) was above 60 years. 52% (n=65) of the nurses had worked for more than 12 years while 4% (n=5) had worked for 2 years and under. About 62.6% (n=77) of them were community health nurse and 29.3 % (n=36) were midwives.

All the nurses in this study understood what screening for DV means, but just a few of them (16%, n=8) routinely screened pregnant woman for DV as shown by Table 1 below.

The situations that prompted the nurses to screen for DV included when pregnant women reported abuse the clinical interview (94.3%, n=82). Also, when they presented with physical indicators of abuse (93.3%, n=83) as shown in Table 2 below.

The results showed that 98% (n=123) of the nurses did not utilize any standard tool while screening for DV as shown in this Table 3 below.

There was a statistical significance between respondent's non-screening behaviour and the respondents lack of DV screening training during their education program (P=0.002), fear of the partner’s reaction (P=0.004) and lack of mentors and role models in DV screening (P=0.005). Lack of cooperation from other health professionals was also correlated with the non-screening DV (P=0.016).

## Discussion

The results of this study revealed that nurses understood what “screening” for DV in pregnancy entails. This results support Hindin [9] and Guruge [21] who reported that nurses and midwives are concerned, interested and knowledgeable about DV screening.

Among the nurses interviewed, only 16% (n=20) routinely screened pregnant women for DV despite their understanding of DV screening. This is low compared to 26% in Nigeria [22] and 50% in Sweden [23] but higher than in Jordan where it was 10% [24]. This could be partly explained by weak health care systems in the developing countries which are yet to embrace the benefits of DV screening [3]. However, this could also be explained by lack of consensus on the frequency of screening [7,16–18].

Globally, there is an agreement that when screening for DV, standard tools should be used [25,26]. In this study, 98% (n=123) of the nurses did not use any standard tool when screening for DV. The respondents reported that they used general question, especially when there were leading cues from the survivors’ history or physical examination findings. Majority used questions like “What caused the physical injury that you have?” This could be as a result of a weak Kenya National Reproductive Health Policy. The policy seeks to ensure access to quality treatment and rehabilitation reproductive health services for survivors of gender-based violence. However, the policy does not provide step by step guidelines and tools to be used in the management of gender-based violence to include DV [27].

A large number of the respondents had worked for 12 years and therefore they were trained some years back and this could explain their response of lack of DV management training. Gender Based Violence may have not been a public health concern then and hence the education system, then emphasized only on the health priorities of those days. However, in the recent past, there has been a rise in the prevalence of DV [28]. This calls for retraining of health workers in DV management. As Boinville [15] documents, there is a positive association between provider training and subsequent adherence to DV management protocols.

Lack of cooperation from other health care professionals was another barrier from this study. There has been a strained relationship between nurses and medical doctors in the recent past. This is a worrying trend since the two health professionals interact during the care of patients. The two cadres (doctors and nurses) have poor communication relations and mistrust on the quality of care provided by each cadre [29]. Also, as East et al. [30] reported, nurse’s autonomy is also affected and limited by the traditional doctor-nurse role hierarchy. It is of the view of the researchers that optimal relationship and multidisciplinary collaborations among all health care providers should be encouraged if routine DV screening is to be achieved.

## Conclusion

The study revealed that prevalence of screening pregnant women for DV is low, standing at 16%. Nursing barriers to screening include: lack of DV screening training during the nursing education program and fear of the partner’s reaction. The barriers also include, lack of mentors and role models in DV screening and lack of cooperation from other health professionals.

## Figures and Tables

**Table 1:** Distribution of respondents screening or non-screening practices.

How often do you currently screen DV among pregnant women?	n	Percentage (%)
Always and Most of the time	20	16
Rarely and Never screen	105	84
Total	125	100%

**Table 2:** Distribution of different situations that respondents screened for DV among pregnant women.

The situations in which nurses screened for DV among pregnant women	Yes	
	n	Percentage (%)
When they are attending hospital appointments	38	42.7
When they are seeking medical care	35	39.3
All new clients	22	25
Clients with physical indicators (physical symptoms) of abuse	83	93.3
Those who report abuse during clinical interview	82	94.3
Only if the client seems distressed	36	41.4
I screen randomly	11	12.6

**Table 3:** Distribution of different screening tools utilized for screening for DV.

Standard tools used when screening pregnant women for DV	No		Yes	
	n	Percentage (%)	n	Percentage (%)
No standard tool	2	1.6	123	98
Hurt, Insult, Threaten and Scream	125	100	0	0
The Woman Abuse Screening Tool	125	100	0	0
The Partner Violence Screen	125	100	0	0
Abuse Assessment Screen	123	98.4	2	1.6
Composite Abuse Scale	125	100	0	0
Conflict Tactics Scale	125	100	0	0
Index of Spousal Abuse	125	100	0	0

## References

[1] WHO (2014). Violence against women: Intimate partner and sexual violence against women.

[2] Shamu S, Abrahams N, Temmerman M, Musekiwa A, Zarowsky C (2011). A systematic review of African studies on intimate partner violence against pregnant women: Prevalence and risk factors. PLoS One.

[3] Undie C, Maternowska C, Mak’anyengo M, Birungi H, Keesbury J (2012). Routine screening for intimate partner violence in public health care settings in Kenya: An assessment of acceptability.

[4] Undie C, Maternowska M, Mak’anyengo M, Askew I (2013). Feasibility of routine screening for intimate partner violence in public health care settings in Kenya.

[5] Chotnopparatpattara P, Limpongsanurak S, Charnngam P (2003). The prevalence and risk factors of anemia in pregnant women. J Med Assoc Thai.

[6] Khasakhala-Mwenesi B, Buluma R, Kong’ani R, Nyarunda V (2007). Gender based violence.

[7] American College of Obstetricians and Gynecologists (2012). Intimate partner violence; Committee Opinion. Obstet Gynecol.

[8] Kothari CL, Rhodes KV (2006). Missed opportunities: Emergency department visits by police identified victims of intimate partner violence. Ann Emerg Med.

[9] Hindin PK (2006). Intimate partner violence screening practices of certified nurse-midwives. J Midwifery Womens Health.

[10] Baig A, Shadigian E, Heisler M (2006). Hidden from plain sight: Residents’ domestic violence screening attitudes and reported practices. J Gen Intern Med.

[11] Coker AL, Smith PH, Bethea L, King MR, McKeown RE (2000). Physical health consequence of physical and psychological intimate partner violence. Arch Fam Med.

[12] Jejeebhoy S, Santhya K, Acharya R (2010). Health and social consequences of marital violence: A synthesis of evidence from India.

[13] Ackerson LK, Subramanian SV (2009). Domestic violence and chronic malnutrition among women and children in India. Am J Epidemiol.

[14] Janssen PA, Holt VL, Sugg NK, Emanuel I, Critchlow CM (2003). Intimate partner violence and adverse pregnancy outcomes: A population-based study. Am J Obstet Gynecol.

[15] Boinville M (2013). ASPE policy brief: Screening for domestic violence in health care settings.

[16] USPSTF (2012). Final recommendation statement: Intimate partner violence and abuse of elderly and vulnerable adults: Screening. Ann Intern Med.

[17] Feder G, Wathen CN, MacMillan HL (2013). An evidence-based response to intimate partner violence: WHO guidelines. JAMA.

[18] National Institute for Health and Care Excellence (2014). Domestic violence and abuse: How health services, social care and the organizations they work with can respond effectively.

[19] Barnett C (2005). Exploring midwives’ attitudes to domestic violence screening. Br J Midwifery.

[20] Gutmanis I, Beynon C, Tutty L, Wathen CN, MacMillan HL (2007). Factors influencing identification of and response to intimate partner violence: A survey of physicians and nurses. BMC Public Health.

[21] Guruge S (2012). Nurses’ role in caring for women experiencing intimate partner violence in the Sri Lankan context. ISRN Nursing.

[22] John A, Lawoko S, Svanström L (2011). Screening for IPV in healthcare in Kano, Nigeria: extent and determinants. J Fam Violence.

[23] Lawoko S, Sanz S, Helstrom L, Castren M (2011). Screening for intimate partner violence against women in healthcare sweden: Prevalence and determinants. ISRN Nursing.

[24] Al-Natour A, Gillespie GL, Felblinger D, Wang LL (2014). Jordanian nurses’ barriers to screening for intimate partner violence. Violence Against Women.

[25] Family Violence Prevention Fund (2004). National consensus guidelines on identifying and responding to domestic violence victimization in healthcare setting.

[26] American Nurses Association (2000). American Nurses Association position statement on violence against women.

[27] Ministry of Health (MOH) (2009). National Reproductive Health Policy enhancing reproductive health status for all Kenyans.

[28] Kenya National Bureau of Statistics (KNBS) and ICF Macro (2010). Kenya Demographic and Health Survey 2008–09.

[29] Foth T, Block K, Stamer M, Schmacke N (2015). The long way toward cooperation: Nurses and family physicians in Northern Germany. Glob Qual Nurs Res.

[30] East LA, Arudo J, Loefler M, Evans CM (2014). Exploring the potential for advanced nursing practice role development in Kenya: A qualitative. BMC Nursing.

